# Development of a High-Sensitivity Acoustic Sensor Based on Grating Interferometer Combined with Glass Diaphragm

**DOI:** 10.3390/mi15091097

**Published:** 2024-08-29

**Authors:** Mengying Zhang, Chao Lu, Quanliang Zhao, Zhi-Mei Qi

**Affiliations:** 1 School of Mechanical and Materials Engineering, North China University of Technology, Beijing 100144, China; zhangmengying411@163.com (M.Z.); lckj18810553509@outlook.com (C.L.); 2 State Key Laboratory of Transducer Technology, Aerospace Information Research Institute, Chinese Academy of Sciences, Beijing 100190, China; 3 College of Materials Science and Opto-Electronic Technology, University of Chinese Academy of Sciences, Beijing 100049, China

**Keywords:** acoustic sensor, grating interferometer, micromachining, squeeze film damping

## Abstract

In this study, based on the principle of grating interferometer-based acoustic sensors, design guidelines for the grating interferometric module were obtained and analyzed considering various factors in order to obtain high sensitivity, and a glass-based grating interference component and its acoustic sensor device were developed. The key parameters of the grating interference structure were extracted, and their effects on the acoustic response sensitivity were quantified for multiple mechanisms. For the development of the acoustic sensor, the grating-on-convex-platform structure and the micromachining processes of the glass-based components were designed and developed. The developed acoustic sensor samples achieved high sensitivity. In particular, the sample suitable for low-frequency application obtained a sensitivity of 0.776 V/Pa @ 1 kHz, and the spectrum of its sensitivity was flat from 50 Hz to 8 Hz with a deviation less than 1.5 dB and a sensitivity of 0.408 V/Pa @ 20 Hz.

## 1. Introduction

Acoustic sensors have been widely applied to environmental monitoring, security monitoring, speech recognition, and mechanical fault diagnosis. Acoustic sensors based on the optical method have the advantages of high sensitivity, efficient multiplexing, and immunity to electromagnetic interference. Especially, the optical interferometric methods are able to measure sub-nanometer displacement [1,2], and they then have the ability to detect weak acoustic signals with sensing structures [3,4]. Because the lower-frequency vibration signals are more suitable for detection by direct displacement measurements, interferometric sensors are commonly used for high-precision, lower-frequency measurements, such as acceleration sensors [5,6], and infrasonic–audible acoustic sensors used for natural disaster monitoring, photoacoustic detection [7], and vocal object positioning [8].

As electrical sensors have been well realized in miniaturization and integration, optical acoustic sensors based on microstructures and integrated optical systems have attracted much attention. Sensors based on micrograting interferometers can achieve high sensitivity and improved stability [9,10] because their accurately fabricated integrated interference structure obtains the coincidence of the interference light field and reduces the loss of the optical path.

For sensors based on grating interferometers, the response to the measured signals is divided into two sections: the sensing structure responds to the measured signals, and the grating interferometer, which is composed of the sensing structure and the grating, responds to the displacement of the sensing structure. Because the displacement measurement with the grating interferometer is a non-contact measurement, the two responses hardly affect each other, and the sensitivity of the whole sensor is the product of the sensitivities of the two responses. More research is focused on improving the performance of the sensing structures, as their design is more flexible. For acoustic sensors, the diaphragm is the most common sensing structure. In addition to the conventional flat diaphragm, microstructures have been designed in the diaphragm to improve its responses to the measured acoustic signals, such as flywheel structures [11,12] and annular corrugations [13]. Limited by conventional micromachining processes, a few kinds of materials have been used to fabricate the diaphragms, such as metal [14], silicon [15,16], glass and silicon carbide [17], and sapphire [18], the last two of which are suitable for high-temperature application. The analyses of the structure design in the reported works mainly focus on the length and thickness of the sensing structure with fixed material, considering its deformation under force loading. For the grating interferometric module, in addition to the research that analyzes the response deviation with the vector diffraction theory [19], most of the research for developing sensors adopts the basic relationship between the interference intensity and the cavity length with ideal, simplified scalar theory [10,20]. Then, the relationship is approximately cosine and is simply considered as linear in a small measurement range. However, this cosine-based sensing relationship is seriously affected by the operating point [21,22]. Temperature is a common factor affecting the stability of the operating point for optical interference sensors [23]. A typical method is to reduce the length of the interference cavity to reduce the drift of the operating point under temperature changes with a short cavity structure [9,24]. However, it is necessary to study and optimize the significant damping effects of the microcavity structure.

In this work, the displacement measurement principle of the grating interferometric module and the response of the diaphragm to acoustic pressure were analyzed separately. Key parameters of the grating interference structure, which affect the sensor’s performance, were extracted, quantified, and optimized with multi-aspect consideration. Acoustic sensors based on the all-glass grating interferometric component were developed, and the samples with different geometrical sizes were analyzed and compared with the sensor samples developed in our preliminary work [9,25]. In addition, the sample with a larger diaphragm obtained high performances for responding to low-frequency acoustic signals.

## 2. Theory and Design Guidelines

An acoustic sensor based on a grating interferometer was developed and analyzed in this study. The theory of the sensor is composed of the response of the grating interferometer to its cavity length and the response of the diaphragm to acoustic pressure.

### 2.1. Theory of Grating Interferometer-Based Sensor

**Figure 1:**
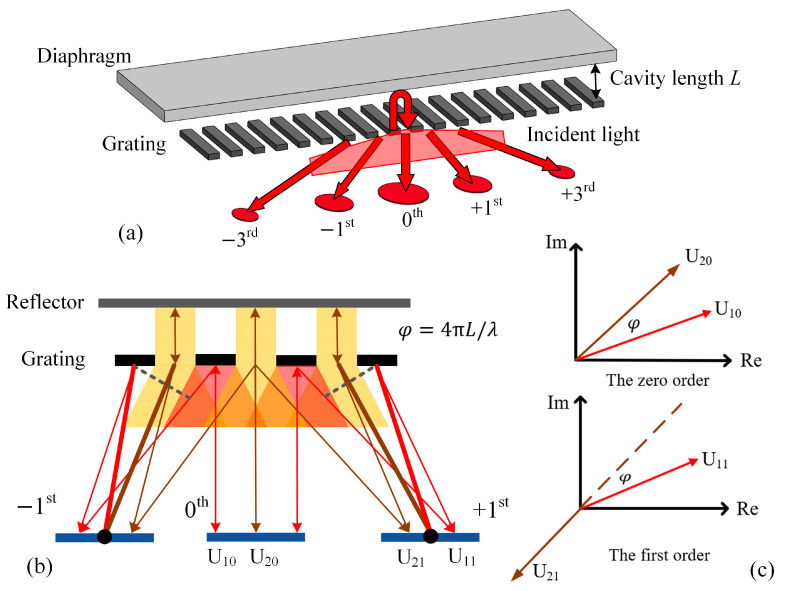
Theory of acoustic sensor based on grating interferometer: (**a**) schematic of the interferometric cavity; (**b**) schematic of the double-grating model for grating interferometer; (**c**) electric field vectors of the light diffracted by the two gratings in the complex plane, where $U_{10}$, $U_{20}$, $U_{11}$, and $U_{21}$ are the electric field vector of the fringe grating at the zero order, the electric field vector of the slit grating at the zero order, the electric field vector of the fringe grating at the first order, and the electric field vector of the slit grating at the first order, respectively.

The theory of the acoustic sensor based on the grating interferometer is shown in Figure 1a, and the interference cavity consists of a diaphragm and a one-dimensional diffraction grating. The vertical distance between the bottom surface of the diaphragm and the grating plane is the length of the interference cavity, represented by *L*. When the coherent light is vertically incident on the grating from outside, the light is diffracted and a part of it is reflected back. The other part that passes through the grating transmits in the interference cavity and is reflected back by the bottom surface of the diaphragm. When the light reaches the grating again, it is diffracted and a part of it passes through the grating again. The two parts of diffracted light interfere in the area where the incident light originates. The enhanced interference light appears at the diffraction orders of the grating, the zero order and odd orders. The phase difference in the interference light is determined by the optical path of the round-trip transmission in the interference cavity. When acoustic pressure is loaded on the diaphragm, it is bent, causing the displacement of its bottom surface, which changes both the cavity length and the interference light. As a result, the output interference light of the grating interferometer is modulated by the acoustic signals.

To analyze the model of the optical interference structure, intensities of the interference light at the zero and odd diffraction orders were derived with scalar diffraction theory. For simplified analysis, the grating-reflector model is regarded as two gratings. One of them comprises the reflective fringes of the grating, referred to as the fringe grating, and the other one comprises transparent slits, referred to as the slit grating, as shown in Figure 1b. According to the mechanism of the grating interferometer, the diffraction light of the fringe grating is the light first reflected by the grating, and the diffraction light of the slit grating is the light transmitted through the grating again after the round-trip transmission in the cavity. The diffraction fields of the two gratings are staggered vertically relative to the fringes, and the staggering length is the distance between the centers of the adjacent fringe and slit, meaning half of the grating period (Λ).

For the interference between the diffraction light fields of the two gratings, there is an initial phase difference of 4πL/λ at the grating plane due to the light path in the cavity, and an additional phase difference that is caused by the staggering distance of the two gratings, which varies with the optical transmission. Considering the zero and first diffraction orders of the grating interferometer, which are usually used as output signals of the sensor, the electric field vectors of the light diffracted by the two gratings are shown in Figure 1c. At the zero order, with a diffraction angle of 0, the phase difference in the two electric field vectors is only φ=4πL/λ. At the first diffraction order, with a diffraction angle of θ=arcsin⁡(λ/Λ), the phase difference in the two electric field vectors is the sum of the initial one (φ) and the additional one:

(1) $$\varphi_{a}=\frac{2\pi \left(\frac{\Lambda }{2}sin\theta \right)}{\lambda }=\pi$$

due to the staggering length between the two gratings being half of the grating period.

According to scalar diffraction theory, the intensities of the interference light at the two orders are as follows:

(2) $$I_{0}={\left|U_{10}+U_{20}\right|}^{2}={\left|U_{10}\right|}^{2}+{\left|U_{20}\right|}^{2}+2\left|U_{10}\right|\left|U_{20}\right|\cos\varphi$$

(3) $$I_{1}={\left|U_{11}+U_{21}\right|}^{2}={\left|U_{11}\right|}^{2}+{\left|U_{21}\right|}^{2}+2\left|U_{11}\right|\left|U_{21}\right|\cos\left(\pi -\varphi \right)\;={\left|U_{11}\right|}^{2}+{\left|U_{21}\right|}^{2}-2\left|U_{11}\right|\left|U_{21}\right|\cos\varphi$$

which indicates that the changes in the interference light intensity with the cavity length at the two diffraction orders are opposite. For simplified analysis, in the model with 50%-duty-cycle grating, the intensities of the two gratings’ diffraction light fields are considered as equal, and then the interference light intensities at the two diffraction orders accord with other reported analyses of grating interferometer-based sensors:

(4) $$\;\left|U_{10}\right|=\left|U_{20}\right|=\frac{\sqrt{I_{in}}}{2}$$

(5) $$\left|U_{11}\right|=\left|U_{21}\right|=\frac{2}{\pi }\left|U_{10}\right|=\frac{\sqrt{I_{in}}}{\pi }$$

(6) $$\;I_{0}=\frac{I_{in}}{2}\left[1+\cos\left(\frac{4\pi L}{\lambda }\right)\right]$$

(7) $$I_{1}=\frac{2I_{in}}{\pi^{2}}\left[1-\cos\left(\frac{4\pi L}{\lambda }\right)\right]$$

where $I_{in}$ is the intensity of the incident light.

The intensities of the two gratings’ diffraction light fields are hardly equal in the actual interferometer due to the loss at the grating fringes and the reflector, and the duty cycle of the grating varying between 0 and 1 [2]. But the opposite relationship between the changes in the interference light intensity at the zero order and the first order is invariant:

(8) $$\Delta I_{0}=A_{0}I_{in}\cos\left(\varphi_{0}+\Delta \varphi \right)$$

(9) $$\Delta I_{1}=-A_{1}I_{in}\cos\left(\varphi_{0}+\Delta \varphi \right)$$

(10) $$\;\varphi_{0}=\frac{4\pi L_{0}}{\lambda },\Delta \varphi =\frac{4\pi \Delta L}{\lambda }$$

where $A_{0}$ and $A_{1}$ are the coefficients of the interference intensity variation at the two orders, decided by the reflectivity of the grating and the reflector, and the duty cycle of the grating. The initial operating point ($\varphi_{0}$) is decided by the initial length of the interference cavity (*L*_0_), and the phase change (Δφ) is decided by the length change of the interference cavity (Δ*L*), which corresponds to the measured displacement.

**Figure 2:**
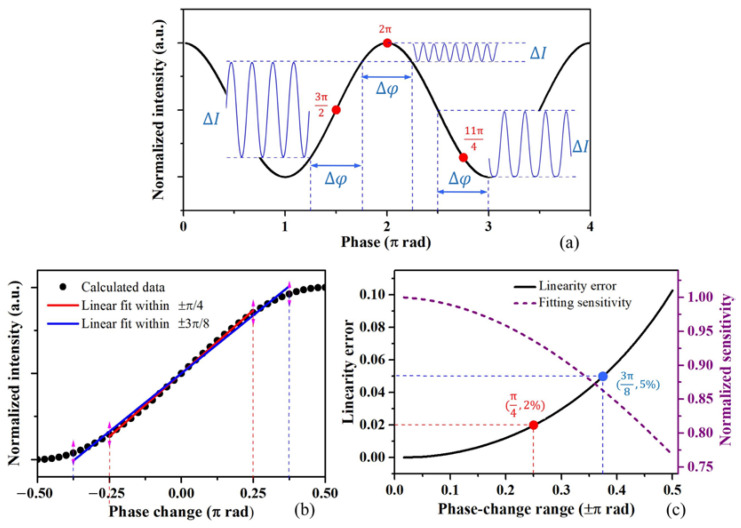
Relationship between the interference light intensity and the interference phase: (**a**) the nonlinear relationship and the effect of the initial operating point, (**b**) the relationship within the phase variation range of ±π/2, with the orthogonal operating point, and (**c**) the linearity error and the sensitivity versus the phase variation range, with the orthogonal operating point.

As a result, at both diffraction orders, the interference intensity varies as a cosine function of the cavity length and the interference phase. As shown in Figure 2a, the nonlinear sensing relationship between the ΔI and Δφ depends on the initial operating point ($\varphi_{0}$). The highest sensitivity and largest linear measurement range are obtained with an orthogonal operating point, $\varphi_{0}=\left(2n+1\right)\pi /2$, where *n* is an integer. Variations in the operating point will change the sensing response and lower the performance of the sensors. As shown in Figure 2a, the deviation of the operating point will lead to serious distortion in the appropriate measurement range, with even-order harmonic components appearing in the light intensity signals. For applications of the sensors based on grating interferometers, the interference phase difference and the measured displacement are usually demodulated from the interference light intensity changes with an approximate linear relationship. Although with the orthogonal operating point, the linear measurement range in the cosine sensing relationship is limited, as shown in Figure 2b. The linearity error and fitting sensitivity versus the phase variation range are shown in Figure 2c, and the two highlighted data points in the graph correspond to the results of the linear fits within the two ranges in Figure 2b, identified by the corresponding colors. It is indicated that the linear error increases while the approximate sensitivity decreases with the measurement range increase. Quantitatively, when the phase range exceeds ±3π/8, the linear error is larger than 5% and rapidly increases, and the error is so large that the linear relationship is not applicable.

### 2.2. Design Guidelines for the Interference Cavity

#### 2.2.1. Static Deformation of the Diaphragm

Except for the basic theory of the grating interferometer, the diaphragm is important for the response to the acoustic pressure. For conventional circular flat film, its deformation under uniform pressure has been sufficiently studied [26,27]. Then the key parameters of the diaphragm in the optical interference acoustic sensor are analyzed based on the theories.

For the common measurement range of the optical interference sensor, as shown in Figure 2c, the interferometric phase variation (Δφ) should be less than 3π/8 for adequate linearity. The laser sources used in the sensors are usually visible light or infrared light, with a wavelength of less than 2 μm. Calculated by Equation (10), the range of the displacement (Δ*L*), which is suitable for linear measurement directly, is less than 0.375 μm. According to the basic theory of the diaphragm’s normal deformation (Δy) under uniform pressure (P), there is the nonlinear term with the ideal linear term, where ${\Delta y}^{3}$ is proportional to the P. The nonlinear term is caused by the stretch of the median plane of the diaphragm when there is a large deformation, and a larger deformation means a larger linearity error in the relationship between the deformation and the pressure. When the maximum deformation is less than 30% of the thickness, the linearity error is less than 2% [26]. As a result, for the diaphragm with a micron thickness, the deformation is approximately linear with the loaded pressure in the measurement range. Then, the deformation of a circular flat diaphragm with a fixed edge under uniform pressure (*P*) is as follows:

(11) $$\Delta y\left(r\right)=\frac{3\left(1-\mu^{2}\right)P}{16Eh^{3}}{\left(R^{2}-r^{2}\right)}^{2}$$

where E and μ are the Young’s modulus and Poisson’s ratio of the material, respectively, R and h are the radius and thickness of the circular diaphragm, respectively, and r is the radial coordinate. For the optical interference sensor, the maximum deformation at the center is approximately considered to be the measured displacement:

(12) $$\;{\Delta y}_{max}=\Delta y\left(0\right)=0.1875K_{m}K_{g}P$$

(13) $$\;K_{m}=\frac{1-\mu^{2}}{E},K_{g}=\frac{R^{4}}{h^{3}}$$

where $K_{m}$ is set as the material parameter of deformation and $K_{g}$ is set as the geometry parameter of deformation. As a result, the deformation of the diaphragm under a certain pressure, meaning the interferometric phase variation in the sensor under a certain acoustic pressure, is linear with the two parameters.

#### 2.2.2. The Incident Light Beam

**Figure 3:**
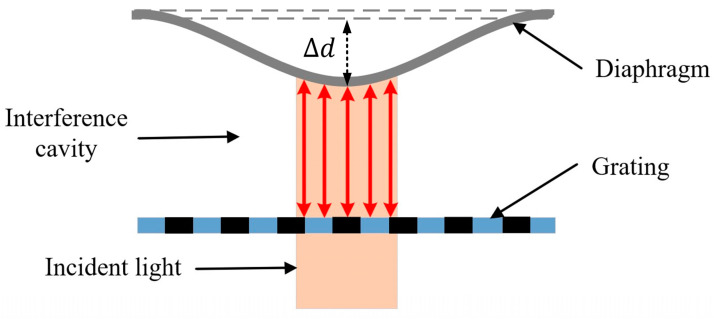
Schematic diagram of the interference cavity with a deformed diaphragm, where the red arrows represent the optical path of the round-trip transmission in the interference cavity.

Only the maximum displacement at the center of the diaphragm is considered in the response of the sensor in the above analysis, the same as in other published articles. For the practical device, the size of the incident light beam affects the total intensity of the output interference light because of the deformation distribution in the diaphragm. For the parallel light beam with a radius of $r_{l}$, the total intensity of the output interference light is the sum of the interference light elements output from the interference cavity elements composed of the grating and the bending diaphragm in the region of the light beam, as shown in Figure 3.

So, the corrected intensity of the output interference light with the radius of the incident light beam is as follows:

(14) $$\Delta I_{n}^{\prime }=A_{n}\int_{0}^{r_{l}}2\pi ricos\left[\varphi_{0}+\frac{4\pi \Delta d\left(r\right)}{\lambda }\right]dr$$

(15) $$\;i=\frac{I_{in}}{\pi r_{l}^{2}}$$

(16) $$\Delta d\left(r\right)=\Delta d_{c}\frac{{\left(R^{2}-r^{2}\right)}^{2}}{R^{4}}$$

where $\Delta I_{n}^{\prime }$ and $A_{n}$ are the variations in the interference intensity and its coefficient at the nth order of the grating interferometer, respectively, i is the areal density of the parallel incident light, $\Delta d\left(r\right)$ is the variation in the cavity length at the radial distance (*r*) off the center, and $\Delta d_{c}$ is the variation at the center, equal to the ${\Delta y}_{max}$ in Equation (12). Then, the equations are simplified with the orthogonal operating point, $\varphi_{0}=\pi /2$:

(17) $$\Delta I_{n}^{\prime }=A_{n}\frac{I_{in}}{K_{l}}\int_{1}^{1-K_{l}}sin\left[\varphi_{c}t^{2}\right]dt$$

(18) $$\;K_{l}=\frac{r_{l}^{2}}{R^{2}}\varphi_{c}=\frac{4\pi \Delta d_{c}}{\lambda }$$

where $K_{l}$ is set as the parameter of incident light. Compared with the conventional result of the interference light as in Equation (19), a correction factor (*q*) is set:

(19) $$\Delta I_{n}=A_{n}I_{in}\cos\left(\frac{\pi }{2}+\varphi_{c}\right)=-A_{n}I_{in}\sin\left(\varphi_{c}\right)$$

(20) $$\;q=\frac{\Delta I_{n}^{\prime }}{\Delta I_{n}}=-\frac{\int_{1}^{1-K_{l}}sin\left[\varphi_{c}t^{2}\right]dt}{K_{l}\sin\left(\varphi_{c}\right)}$$

**Figure 4:**
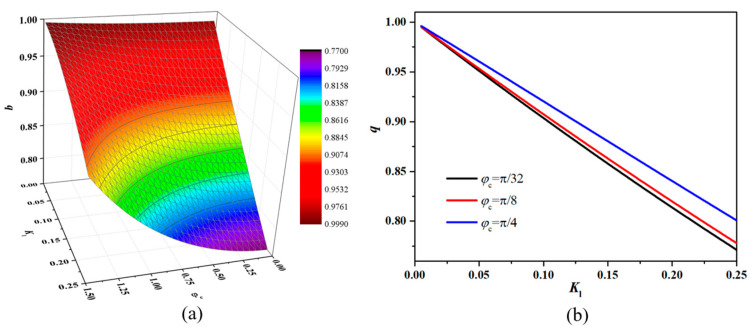
Calculation results of the correction factor (*q*) by MATLAB: (**a**) 3D surface graph with parameters $K_{l}$ and $\varphi_{c}$; (**b**) relationships between the factor (*q*) and parameter $K_{l}$ with a certain $\varphi_{c}$.

The Fresnel sine integral in the equations is hard to derive directly, so the factor (*q*) is calculated by software MATLAB R2013b. As shown in Figure 4, the factor (*q*) decreases with the increase in the parameter $K_{l}$, and there is an approximately linear relationship within the conventional range. This means that the amplitude of the interference light signal responding to the acoustic pressure will be obviously weakened when the diaphragm in the sensor is not large enough compared with the incident light.

#### 2.2.3. Squeeze Film Damping

As the acoustic sensors mostly work in gas or liquid, the influence of the squeeze film damping on the vibrating diaphragm under acoustic pressure is nonnegligible. Although air damping is much smaller compared to liquids, it will become very large when the length of the interference cavity between the intact planes of the diaphragm and the grating is quite small for the MEMS sensors.

According to the analysis for the model of a thin flat film of an ideal gas with surfaces bounding in relative normal motion [28,29], the damping force on the unit area of the gas film is as follows:

(21) $$\;\bar{F}=-\frac{P_{a}}{d_{a}}f_{e}\left(\sigma \right)y-\frac{P_{a}}{d_{a}\omega }f_{v}\left(\sigma \right)\dot{y}$$

(22) $$y=y_{0}cos\omega t$$

where $P_{a}$ is the pressure of the gas, $d_{a}$ is the initial thickness of the gas film, y means the normal displacement of the gas film’s surfaces, and ω is the angular frequency of the displacement. The damping force consists of the elastic damping force proportional to the displacement and the viscous damping force proportional to the velocity of the displacement, the two parts in Equation (21). The coefficients, $f_{e}\left(\sigma \right)$ and $f_{v}\left(\sigma \right)$, are determined by the squeeze number (σ):

(23) $$\;\sigma =\frac{12\mu_{a}\omega R_{a}^{2}}{P_{a}d_{a}^{2}},k=\sqrt{\frac{\sigma }{2}}$$

(24) $$\;f_{e}\left(\sigma \right)=1-\frac{1}{k}\frac{sinhk+sink}{coshk+cosk}$$

(25) $$f_{v}\left(\sigma \right)=\frac{1}{k}\frac{sinhk-sink}{coshk+cosk}$$

where $\mu_{a}$ is the viscosity of the gas, and $R_{a}$ is the radius of the gas film. To simplify the analysis for the infrasonic–audible acoustic sensor in gas ambient, the two coefficients are replaced as follows:

(26) $$f_{e}\left(\sigma \right)=\frac{\sigma^{2}}{120},f_{v}\left(\sigma \right)=\frac{\sigma }{12}$$

and their deviations are less than 16% when the σ is less than 4 for most of the MEMS acoustic sensors detecting acoustic singles under 10 kHz, as shown in Appendix A. For the sensor based on a grating interferometric module with an extremely short cavity [30], the high-perforation-ratio grating substrate is adopted to reduce the squeeze film damping.

For the basic structure of the grating interferometer consisting of a flat diaphragm and grating, the initial thickness ($d_{a}$) and radius ($R_{a}$) of the gas film in Equation (23) are equivalent to the initial cavity length ($L_{0}$) and radius of the diaphragm (R), respectively. Then, the damping force is derived as follows:

(27) $$\bar{F}=-\frac{1.2\mu^{2}\omega^{2}}{P_{a}}K_{d-e}y-\mu_{a}K_{d-v}\dot{y}$$

(28) $$\;K_{d-e}=\frac{R^{4}}{L_{0}^{5}},K_{d-v}=\frac{R^{2}}{L_{0}^{3}}$$

(29) $$k_{d}=\frac{1.2{\mu_{a}}^{2}\omega^{2}}{P_{a}}K_{d-e},c_{d}=\mu_{a}K_{d-v}$$

where $K_{d-e}$ is set as the parameter of elastic damping and $K_{d-v}$ is set as the parameter of viscous damping. For the acoustic sensor operated in air, the two parameters mean the influence of its structure on the damping force loading on the vibrating diaphragm.

Furthermore, considering the vibrating diaphragm under acoustic pressure as the spring–damper model, the elastic damping coefficient ($k_{d}$) should be added with the elastic coefficient for the deformation of the diaphragm, and the viscous damping coefficient $c_{d}$ should be added with the damping coefficient, both of which will reduce the vibration amplitude of the diaphragm loaded with a certain acoustic pressure.

#### 2.2.4. The Key Parameters

According to the above discussions, the key parameters of the interference cavity structure that decide the characteristics of the diaphragm deformation under acoustic pressure are shown in Table 1.

Unlike the limitations of the materials due to the micromachining processes, the design of the geometric parameters is much more flexible. The parameters in Table 1 indicate that the radius of the flat diaphragm is the most important parameter, compared to the thickness of the diaphragm and the radius of the incident light. A larger radius means larger deformation of the diaphragm and a larger amplitude of the interference signals. Although the larger radius means larger squeeze film damping, appropriately increasing the cavity length properly will reduce the damping effect significantly because the index of the cavity length is higher in the parameters for damping. If the parameters for damping are small enough to make the coefficients of air damping much less than the elastic coefficient of the diaphragm deformation, then the damping effect is negligible.

## 3. Design and Fabrication of the Acoustic Sensor

An acoustic sensor consisting of a glass-based interference cavity component and the board-level optoelectronic module were designed and fabricated in this study.

### 3.1. Structure of the Acoustic Sensor

**Figure 5:**
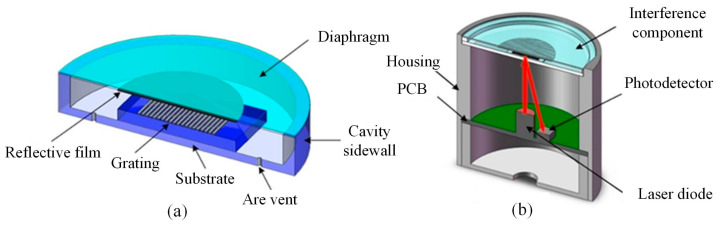
Structure of the acoustic sensor: (**a**) the glass-based interference component, and (**b**) the packaged device with the optoelectronic module, where the red arrows represent the optical path of the incident light and the received light.

The structure of the acoustic sensor is shown in Figure 5. As shown in Figure 5a, the glass-based interference component is composed of a diaphragm, a substrate with the grating, and a cavity sidewall. There is a reflective film on the bottom surface of the glass diaphragm for higher reflectivity. A smaller transparent plate with one-dimensional metallic grating is assembled on the bottom substrate of the component to compose a grating-on-convex-platform structure. The diaphragm is assembled on the substrate with the cavity sidewall, and the center of the diaphragm and the vertically corresponding grating constitute the interference cavity. The grating-on-convex-platform structure is designed to obtain a shorter interference cavity and smaller squeeze film damping. There are air vents in the substrate around the grating platform to eliminate the influence of the slow fluctuation of the ambient pressure, but several small air vents hardly affect the squeeze film damping for higher-frequency responses.

As shown in Figure 5b, the interference component is packaged on the top of a housing with a board-level optoelectronic module assembled under it. For the optoelectronic module, a laser diode and photodetector are installed in the printed circuit board (PCB) with a matched optoelectronic readout circuit. The laser diode provides the light source for the grating interferometer. It is installed at the center and vertically under the interference component. The photodetector is used to detect the reflected interference light of the grating’s first diffraction order. It is installed beside the laser diode with a specific distance, which is decided by the first-order diffraction angle and the distance from its surface to the grating surface in the interference component. The optoelectronic readout circuit contains a supply unit, transconductance unit, and filtering unit for driving the laser diode and conditioning signals from the photodetector. There is an interface at the bottom of the housing to lead out the leads of the optoelectronic module.

### 3.2. Fabrication of the Device

**Figure 6:**
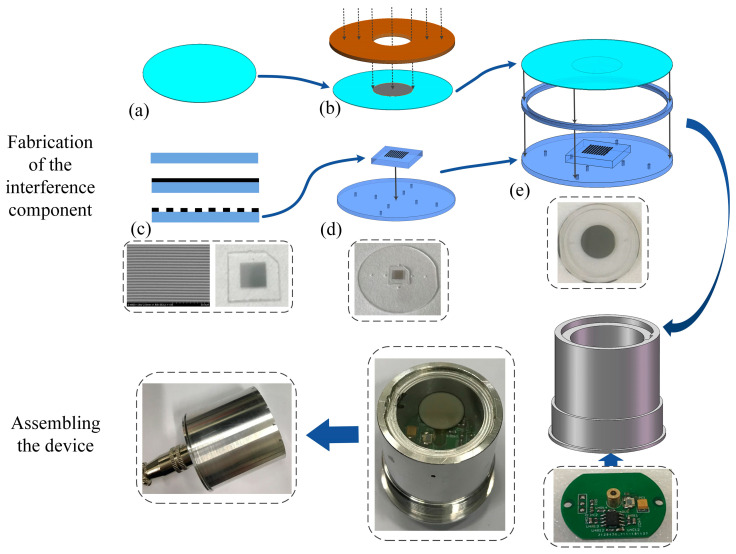
The fabrication process of the acoustic sensor, with photos of the fabricated samples in dashed frames.

The fabrication process of the acoustic sensor is shown in Figure 6. The interference component was mainly fabricated by the micromachining process and laser process, and it was then packaged on the housing with the assembled optoelectronic module.

First, the interference component was fabricated with borosilicate glass substrates, and the process was as follows:

(a) The round diaphragm with the designed diameter was cut by a laser process from the thin glass substrate with the designed thickness. The laser process was direct-writing ablation by the focused laser beam with a 355 nm wavelength and 15 W power, and it was implemented by laser engraving equipment. The direction of the laser beam is adjusted according to the control program, focusing the laser spot on the surface of the substrate moved, while removing its material within a certain depth in the scanned area with high temperature. The laser process is mainly applied for cutting structures from the glass substrates in this fabrication process;
(b) The 100 nm thick chromium (Cr) film was sputtered on the center of the diaphragm with a patterned glass plate covering its surface as the mask;
(c) The grating with a 2.4 μm period was made by the micromachining process flow for preparing high-accuracy metal microstructures on the substrate. First, the 50 nm thick Cr film was sputtered on the glass wafer. Second, a photoresist mask containing a grating fringe pattern was obtained via a lithography process. Third, the Cr film was patterned by high-precision ion beam etching (IBE) processes with the photoresist mask. Finally, discrete square grating plates were cut from the fabricated wafer by the laser process, with the edges parallel or perpendicular to the grating fringe and a missing angle for marking the fringe direction;
(d) The round substrate with the designed positioning edge and air vents were cut from another glass substrate by the laser process. Then, the grating plate was assembled on the center of the substrate by a low-temperature glass bonding process, with one of its edges parallel to the positioning edge of the substrate;
(e) The glass diaphragm with reflective film, a glass ring cut by the laser process as the cavity sidewall, and the substrate with a grating were aligned and assembled by a low-temperature glass bonding process to obtain the glass-based interference component.

Then, the optoelectronic module was made on the PCB. Except for the matched circuit, a laser diode with an integrated collimation module to obtain a parallel beam of a 650 nm wavelength was installed at the center, and a photodetector matched with the laser was installed beside the laser diode to receive the interference light.

Finally, the fabricated interference component was packaged on the top of the stainless-steel housing, and the optoelectronic module was assembled under it from the bottom of the housing. In addition, an external housing with sound pick-up holes was assembled over the interference component to provide protection. The leads for supply and signal connection were drawn from the bottom of the housing through an interface. The external dimensions of the integrated device are ∅38 mm×36 mm.

## 4. Results

### 4.1. The Test Methods

For testing the performances of the fabricated sensors, assigned acoustic signals were generated from a speaker driven by a waveform generator. The output voltage signals from the sensors were detected by a mixed-signal oscilloscope. The low-frequency characteristic of the fabricated sensors was detected by an acoustic sensor calibration system consisting of an input/output generator module (B&K Nærum, Denmark, 3160-A-042), a phase calibrator (GRAS, Holte, Denmark, 42AE), a calibration microphone (B&K, 4193-L-004), and the measurement software (B&K, PULSE LabShop 21.0.0.671).

### 4.2. Response to the Acoustic Pressure

Two glass-based sensor samples with diaphragms of different geometric parameters were fabricated, as shown in Table 2. For the designed convex-platform grating, the height of the cavity sidewall is 500 μm and the size of the convex platform is 8 mm×8 mm×280 μm, so the distance between the central part of the diaphragm and the surface of the convex platform is 220 μm, meaning that the length of the interference cavity is 220 μm. When analyzing the squeeze film damping, it is reasonable to consider only the central part of the air film in the cavity with the size of 8 mm×8 mm×220 μm because the surrounding damping with a cavity length of 500 μm is much smaller. Then, the damping coefficients are 7.48 $\times 10^{3}\;Pa/m$ and 26.85 Pa·s/m for both samples, which are smaller than those in Table 2.

**Figure 7:**
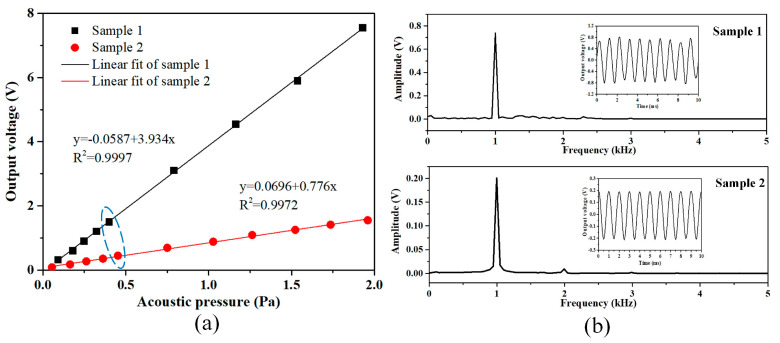
Response of the sensor samples to acoustic signals of 1 kHz frequency: (**a**) relationship between the peak-to-peak values of the voltage of the output signals and the measured acoustic pressures, and (**b**) frequency spectrum of the output signals corresponding to the circled data points in Figure 7a, with the time-domain waveform displayed beside each spectrum.

The responses of the sensor samples to acoustic signals of 1 kHz frequency are shown in Figure 7. As shown in Figure 7a, highly linear relationships were obtained by both the sensor samples within the measured acoustic pressure range of less than 2 Pa. The sensitivity of sample 1 is obviously higher than that of sample 2 (3.934 V/Pa and 0.776 V/Pa, respectively). Furthermore, the frequency spectrum and the time-domain waveform of the output signal of each sample are displayed in Figure 7b, corresponding to the circled data points in Figure 7a with similar loaded acoustic pressure. There is the more obvious peak of the second harmonic in the frequency spectrum of sample 2, which indicates that its operating point deviates from the orthogonal one and the obtained sensitivity is lower than the ideal one, as discussed in Section 2.1.

According to the parameters in Table 2, the elastic coefficient of sample 1 is smaller than that of sample 2, meaning that larger deformation of sample 1 will be obtained under a certain pressure, which is consistent with the measurement results. However, the damping coefficients of the two samples are much smaller than their elastic coefficients, so the influence of the squeeze film damping is negligible.

Furthermore, a micro-acoustic sensor with a silicon-based diaphragm was developed in the previous work [9], and its parameters are listed in Table 2. The interference component in this sensor is composed of a thinner diaphragm made with a silicon substrate and grating made on a flat glass substrate, which is more suitable for micro-integrated sensors. The main differences between the integrated interference component and the one in the glass-based sensor samples are the material and the size of the diaphragm, as well as the very short length of the interference cavity. The sensitivity of the silicon-based sample for 1 kHz acoustic pressure is measured as about 0.2 V/Pa, with the same parameters as the optoelectronic module. Although, its elastic coefficient is much lower than that of the glass-based samples due to the significantly reduced thickness of the silicon diaphragm. Its damping coefficients are even larger than its elastic coefficient because of its short-cavity structure for temperature stability. As a result, the sensitivity of the silicon-based sample was significantly reduced by the effect of squeeze film damping. In addition, the parameter for incident light in the silicon-based sample was relatively larger, and there was a minor effect on its sensitivity, according to Figure 4b.

### 4.3. Low-Frequency Characteristic of the Glass-Based Sensor

The low-frequency characteristic of the fabricated sensor sample 2 with a larger diaphragm was tested by the acoustic sensor calibration system. The generator in the system drove the calibrator to provide sweep acoustic signals within the frequency range from 50 Hz to 0.1 Hz. The output signals of the sensor sample and the calibration microphone were then detected and processed. The sensitivity of the sensor at each frequency was calculated using the measurement software by comparing the output voltage of the sensor with one of the calibration microphones with known sensitivity.

**Figure 8:**
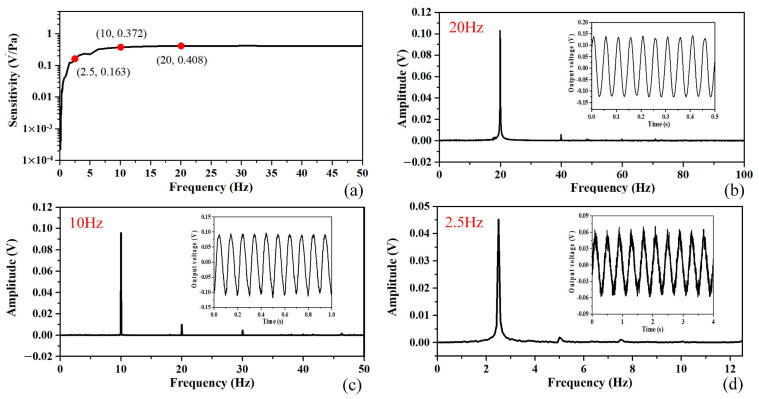
Frequency response of the fabricated sensor sample 2 at low frequency: (**a**) sensitivities changing with the frequencies of measured acoustic pressure within the frequency range from 50 Hz to 0.1 Hz, with the coordinate values of the marked key data points, which are at the demarcation frequency of the infrasound, 20 Hz, and at half and one-eighth of the demarcation frequency, 10 Hz and 2.5 Hz, respectively; (**b**–**d**) frequency spectra of the output signal at 20 Hz, 10 Hz, and 2.5 Hz, respectively, with the time-domain waveform displayed beside each spectrum.

The measurement results are shown in Figure 8, demonstrating that the spectrum is flat from 50 Hz to 8 Hz with a deviation of less than 1.5 dB. Three data points are marked in red in the response spectrum and the coordinate is displayed beside each point, representing the values of its frequency and sensitivity. The sensitivities at 20 Hz and 10 Hz are 0.408 V/Pa and 0.372 V/Pa, with 0.88 dB reduction. Then, it decreases to −8 dB at 2.5 Hz, with a sensitivity of 0.163 V/Pa. The frequency spectrum and the time-domain waveform of the output signal at 20 Hz, 10 Hz, and 2.5 Hz are shown in Figure 8b, Figure 8c, and Figure 8d, respectively. There is also an obvious peak of the second harmonic in each frequency spectrum, which is the same as its response at 1 kHz shown in Figure 7b. The acoustic signal of each frequency was detected. As shown in Figure 8d, for the acoustic signal of 2.5 Hz frequency and 0.275 Pa pressure, the signal-to-noise ratio of the output voltage signal is 40 dB.

## 5. Discussion

The response of the acoustic sensor based on the grating interferometer consists of two series parts: the response of the grating interference module to the displacement and the response of the sensitive diaphragm to the acoustic pressure. In addition to the preliminary and approximate derivation of the principle of the grating interference module by the method combining the double-grating model and electric field vector superposition, quantized design guidelines for the interference structure were obtained for the high deformation of the diaphragm and the high optical measurement efficiency of its displacement with multi-aspect consideration. As shown in Table 1, the key parameters for static deformation, incident light dispersion, and squeeze film damping were derived and extracted. Based on these parameters and the relationships between them and their effects on the sensitivity of the sensor responding to acoustic pressure, the structure design of the grating interferometric module and the analysis of its sensing performance become simple and efficient.

The developed acoustic sensors with glass-based grating interference components were tested. The responses of the sensor samples with different sizes of diaphragms to acoustic pressure of 1 kHz were compared. They both obtained high linearity and high sensitivities (3.934 V/Pa and 0.776 V/Pa, respectively). The difference was analyzed mainly with the static deformation parameters, as the other two factors are negligible for their structural sizes. Furthermore, the sensor based on the integrated interference component containing a silicon diaphragm in our previous work was compared with the glass-based ones in this work, based on the proposed design guidelines. Its sensitivity, about 0.2 V/Pa @ 1 kHz, is much lower than what should be achieved only considering the static deformation parameters of the diaphragm. Its short-cavity structure makes the squeeze film damping become the main factor, and the damping coefficient is even higher than the elastic coefficient for static deformation. The comparison of the three sensors with different structural parameters shows that the extracted key parameters of multiple factors can be applied for sensor structure optimization and performance analysis efficiently and concisely. The compact interference cavity structures are usually applied in these kinds of integrated grating interference sensing components. It is necessary to focus on reducing the effect of the squeeze film damping from the thin air layer in the cavity, such as by manufacturing a porous grating backplane [30,31,32], or by designing a moderate cavity length applying the key parameters for damping provided in the design guidelines. In this work, the grating-on-convex-platform structure in the designed glass-based grating interference component not only shortens the length of the interference cavity but also greatly reduces the squeeze film damping of the cavity with a large diaphragm. In addition, this kind of micro-assembled structure design of the grating interference components is suitable for implementing more flexible adjustments of the interference structures to combine more functional structures and optimize the performances of the sensors [21].

The glass-based sensor with a larger glass diaphragm of 25.4 mm diameter, which is suitable for low-frequency application, is the focus of this work, and its characteristic of responding to acoustic signals at low frequencies is the excellent performance newly obtained in this work. The flat spectrum of the sensitivity was obtained from 50 Hz to 8 Hz with a high sensitivity of 0.408 V/Pa @ 20 Hz. And high sensitivity and a high signal-to-noise ratio were still achieved at the lower frequency of 2.5 Hz. However, compared with the glass-based sensor sample 1 with the smaller diaphragm, it has greater application limitations at higher frequencies. Based on the resonance theory of the circular flat diaphragm with a fixed edge [26], the fundamental resonance frequency of the diaphragm of sample 2 is 2.5 kHz, and that of sample 1 is 3.4 kHz. Except for targeted high-sensitivity applications at the resonant frequency, the measurement frequency of broadband acoustic sensors is usually lower than the fundamental resonant frequency of their diaphragms. In addition to the material parameters, the fundamental resonant frequency of the circular flat diaphragm is decided by the ratio of its thickness to the square of its radius. This parameter for dynamic performance is not the content of the design guidelines in this work.

There are two main drawbacks of the current design of the glass-based grating interferometric module, which can be improved in future work. The first is the measurement limitation at higher frequencies, which is mentioned above. The performances of the diaphragm responding to acoustic pressure will be improved by a more microstructural design. The developed fabrication process of the glass-based interference component facilitates the realization of the diverse structure design of the diaphragm [32]. The second is the problem of the initial interference cavity length and the operating point, which have been discussed based on the output signals of sensor sample 2. Existing glass substrates were directly applied in the fabrication process of the interference component. The thickness of each layer is completely decided by the thickness of the glass substrate, especially for the interference cavity. The operating point deviation problem is hard to avoid, and the sensing performance will be seriously reduced, as is discussed in Section 2.1. In future work, the addition of a cavity length modulation structure in the interference component will be considered. The assembling structure of the designed grating interferometric module is convenient for adding more optimization designs.

## 6. Conclusions

In this work, a high-sensitivity acoustic sensor based on a glass grating interferometric module was developed. First of all, the design guidelines for the grating interferometer-based acoustic sensor were obtained for high-sensitivity sensors, considering the response of the grating interferometric module to displacement and the response of the sensitive diaphragm to acoustic pressure. The key parameters for static deformation, incident light dispersion, and the squeeze film damping of the grating interferometric module were derived and extracted to support efficient structural design and performance analysis. Then, a glass-based grating interference component with the grating-on-convex-platform structure was designed, and its micromachining processes were developed. The fabricated grating interference component was packaged in a housing integrated with the photoelectric module for the acoustic sensor device, whose diameter and height are 38 mm and 36 mm, respectively. The developed sensor samples with glass-based grating interference components obtained high sensitivity and linearity at 1 kHz. The sensor sample with a larger glass diaphragm, which is suitable for low-frequency application and is the focus of this work, obtained sensitives of 0.776 V/Pa @ 1 kHz and 0.408 V/Pa @ 20 Hz. Its spectrum of sensitivity was flat from 50 Hz to 8 Hz, and high sensitivity and a high signal-to-noise ratio were still achieved at the lower frequency of 2.5 Hz.

The structure design of the glass-based grating interference component and its micromachining process developed in this work provide support for the flexible design of sensors based on the grating interferometer to improve performances. In future work, thermal expansion coefficients and heights of the convex platform and the cavity sidewall will be matched to keep the cavity length between the diaphragm and the grating constant when the temperature changes.

## Figures and Tables

**Table 1:** The key parameters of the interference cavity structure.

Mechanism	Parameter	Expression	Effect
Deformation	$K_{m}$	$(1-\mu^{2})/E$	Proportional to displacement
	$K_{g}$	$R^{4}/h^{3}$	Proportional to displacement
Light dispersion	$K_{l}$	$r_{l}^{2}/R^{2}$	Proportional to reduction in interference intensity variation
Squeeze film damping	$K_{d-e}$	$R^{4}/L_{0}^{5}$	Proportional to elastic damping
	$K_{d-v}$	$R^{2}/L_{0}^{3}$	Proportional to viscous damping

**Table 2:** Parameters of the sensor samples.

Parameter	Expression	Sample 1	Sample 2	Silicon-Based Sample [9]
Diaphragm radius	R	6.35 mm	12.7 mm	3 mm
Diaphragm thickness	h	50 μm	150 μm	3 μm
Cavity length ^a^	$L_{0}$	220 μm	220 μm	17 μm
Incident light radius	$r_{l}$	0.5 mm	0.5 mm	0.5 mm
Elastic coefficient for deformation	$\frac{1}{0.1875K_{m}K_{g}}$	2.93 × 10^7^ Pa/m	4.95 × 10^7^ Pa/m	2.99 × 10^5^ Pa/m
Parameter for incident light	$K_{l}$	0.0062	0.0016	0.028
Damping coefficients ^b^ $(\frac{\omega }{2\pi }=10\;kHz$)	$\frac{1.2{\mu_{a}}^{2}\omega^{2}}{P_{a}}K_{d-e}$	4.73 × 10^4^ Pa/m	7.6 × 10^5^ Pa/m	8.58 × 10^8^ Pa/m
	$\mu_{a}K_{d-v}$	6.78 Pa·s/m	270.29 Pa·s/m	32,757 Pa·s/m

^a^ The cavity length is the distance between the bottom surface of the diaphragm and the top surface of the convex-platform grating for the glass-based samples. ^b^ The damping coefficients were calculated with the diaphragm radii and cavity lengths in the table.

**Figure A1:**
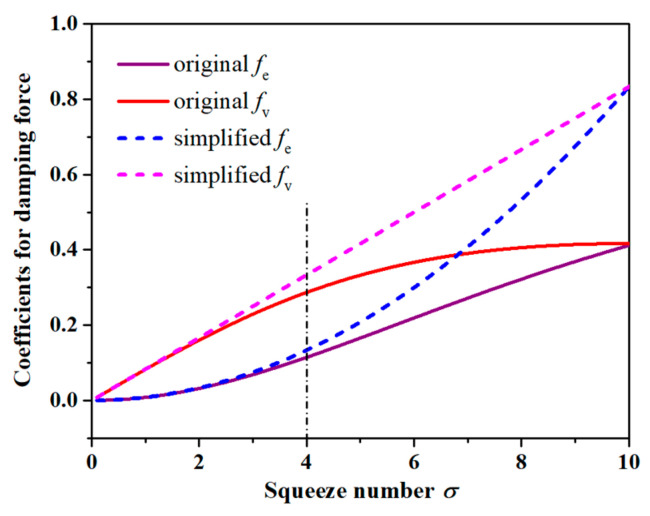
Changes in the coefficients for the damping force with the squeeze number.

## Data Availability

The data that support the findings of this study are available from the authors upon reasonable request.

## Appendix A

As in Equation (23) in Section 2.2.2, the squeeze number is decided by the gas ambient, the vibration frequency, and the geometric parameters. For simplification, using the parameters of standard air and the maximum frequency of 10 kHz, the squeeze number is as follows:

(A1) $$\sigma =1.336\times 10^{-4}\frac{R_{a}^{2}}{d_{a}^{2}}=1.336\times 10^{-4}\frac{R^{2}}{L_{0}^{2}}$$

For most of the reported microsensors based on the grating interferometer with the conventional flat grating structure, the ratio between the radius (R) and the cavity length ($L_{0}$) is less than 176, which was adopted in the short-cavity structure in our previous work [9], meaning that the squeeze number (σ) is about 4.

As in Equations (24)–(26), the coefficients for the damping force, $f_{e}\left(\sigma \right)$ and $f_{v}\left(\sigma \right)$, are shown in Figure A1. When the squeeze number is less than 4, the deviations between the original expressions and the simplified ones are less than 16%.
